# Curcumin as a Natural Remedy for Atherosclerosis: A Pharmacological Review

**DOI:** 10.3390/molecules26134036

**Published:** 2021-07-01

**Authors:** Laxman Singh, Shikha Sharma, Suowen Xu, Devesh Tewari, Jian Fang

**Affiliations:** 1 Centre of Biodiversity Conservation & Management, G.B.Pant National Institute of Himalayan Environment, Almora 263643, Uttarakhand, India; laxmansingh_13@yahoo.com; 2 School of Pharmaceutical Sciences, Lovely Professional University, Phagwara 144411, Punjab, India; shikhasharma22012@gmail.com; 3 Department of Endocrinology, Division of Life Sciences and Medicine, The First Affiliated Hospital of USTC, University of Science and Technology of China, Hefei 230037, China; 4 Department of Pharmacy, Huadu District People’s Hospital, Southern Medical University, Guangzhou 510800, China

**Keywords:** curcumin, atherosclerosis, pharmacology, therapeutics

## Abstract

Curcumin, a natural polyphenolic compound present in *Curcuma longa* L. rhizomes, shows potent antioxidant, anti-inflammatory, anti-cancer, and anti-atherosclerotic properties. Atherosclerosis is a comprehensive term for a series of degenerative and hyperplasic lesions such as thickening or sclerosis in large- and medium-sized arteries, causing decreased vascular-wall elasticity and lumen diameter. Atherosclerotic cerebro-cardiovascular disease has become a major concern for human health in recent years due to its clinical sequalae of strokes and heart attacks. Curcumin concoction treatment modulates several important signaling pathways related to cellular migration, proliferation, cholesterol homeostasis, inflammation, and gene transcription, among other relevant actions. Here, we provide an overview of curcumin in atherosclerosis prevention and disclose the underlying mechanisms of action of its anti-atherosclerotic effects.

## 1. Introduction

Atherosclerosis is a common cause of cerebro-cardiovascular disease and is an age-related chronic large-artery condition that develops in adult and aged patients [1]. The pathogenesis of atherosclerosis is multifaceted. Numerous investigations have highlighted hyperlipidemia, diabetes, smoking, hypertension, and other cardiovascular risk factors which mediate oxidative stress causing damage to vascular endothelial cells. They also cause infiltration of low-density lipoproteins (LDL) into the sub-endothelial space, monocyte chemotaxis, aggregation below the endothelium, and platelet activation leading to chronic inflammatory responses in vascular walls [2,3,4,5]. Atherosclerosis is the pathological basis for many cerebro-cardiovascular diseases and acute cerebro-cardiovascular events such as myocardial infarction and ischemic stroke, making it a serious public health concern [6,7]. Anti-arteriosclerotic traditional Chinese medicines (TCM) are widely used in Chinese clinical practice with a good safety profile and lasting efficacy [8,9]. Many traditional medicines used in TCM and other traditional medicine systems such as Ayurveda including turmeric and ginseng have anti-atherosclerotic effects [10,11].

Turmeric prepared from the dried rhizomes of *Curcuma longa* L. (family, Zingiberaceae) is enriched with multiple bioactive chemical entities with multiple therapeutic applications. The roots and rhizomes of turmeric contain curcumin that has been used as a traditional drug to increase blood circulation and improve stasis [12]. Curcumin has lipid-lowering, antioxidative, anti-inflammatory, and anti-infective effects [13,14,15]. There is growing evidence that curcumin can regulate different signaling molecules to retard the progression and development of atherosclerosis [16]. Similarly, curcumin is also known to regulate inflammatory responses by inhibiting nuclear factor kappa B (NF-κB) expression in atherosclerotic plaques of aortic walls in domestic rabbits and alleviate the severity of atherosclerosis [16].

The mechanistic function of curcumin against atherosclerosis is due at least in part to its anti-inflammatory and anti-oxidative effects and inhibition of vascular smooth muscle cell (VSMC) proliferation and migration. Firstly, inflammation is involved in the entire process of atherosclerosis [17]. According to previous research, curcumin affects inflammatory cells and factors such as inflammation-related enzymes to carry out its anti-inflammatory effects [18,19]. Likewise, curcumin blocks NF-κB signaling to diminish the production of vascular cell-adhesion molecules and inhibit interactions between leukocytes and endothelial cells [20]. Secondly, oxidative stress is a prominent hallmark phenomena that initiates the development of atherosclerosis [21]. Oxidized low-density lipoprotein (oxLDL) is the common link in various aspects of atherosclerosis [22]. Curcumin decreases the sensitivity of LDL towards oxidization, and thus decreases the load of oxidized product to interact with the oxidized low-density lipoprotein receptor 1 (LOX-1) [23]. Curcumin also down regulates inducible nitric oxide synthase activity to inhibit nitro-/oxidative-stress [24]. Thirdly, VSMC proliferation and migration of cells to the intima causes intimal thickening in atherosclerosis. Specifically, neointimal responses associated with artery damage cause proliferation, migration, and collagen synthesis in VSMCs that may increase the susceptibility of blood vessels towards atherosclerosis [25]. Curcumin can increase PPAR-γ activity to inhibit the proliferation of VSMCs [26].

Additionally, epidemiological studies highlight that human cytomegalovirus (HCMV) infection is intimately coupled with the progression and development of atherosclerosis [27]. After entry, HCMV can damage vascular endothelial cells and alter their proliferation [28]. Oral administration of curcumin in ApoE^−/−^ mice inhibits HCMV infection and improves the cellular microenvironment in the host, thereby effectively preventing the development of atherosclerotic lesions [29].

## 2. Atheroprotective Effects of Curcumin In Vitro

The potential of curcumin in protecting against various medical ailments, including atherosclerosis, has been widely assessed. Atherosclerosis is a chronic inflammatory disease resulting from arterial wall injury, sustained due to dyslipidemia, diabetes, hypertension, and other cardiovascular risk factors that leads to macrophage and VSMC-derived foam cell formation, endothelial cell dysfunction, immune cell activation, platelet activation, and thrombus formation [30,31,32,33]. Several studies have demonstrated curcumin’s potent therapeutic potential in preventing foam cell formation, modulating macrophage polarization, tuning cholesterol efflux, and regulating pro-inflammatory responses [16,34,35,36,37,38].

The anti-atherosclerotic properties of curcumin are expressed through suppressing macrophage polarization (M1 to M2) [39] or by inducing M2 polarization via IL-4 and/or IL-13 secretion in macrophages [40]. Similarly, convincing evidence suggests that curcumin, when acting against macrophages treated with oxLDL, upregulates the expression of thrombospondin-4 (THBS-4) [36] and modulates chemoattractant protein-1 (MCP-1) expression, which represents the anti-inflammatory response [41]. The molecular targets of anti-atherosclerotic effects of curcumin involve upregulation of miR-126, which further inhibits signal transduction and PI3K/AKT and JAK2/STAT5 activation [42]. Other targets of curcumin include NF-κB inhibition in the M1 macrophages, as well as promoting M2 phenotype via PPAR-γ activation. Further, curcumin inhibits toll-like receptor-4 (TLR4), MAPK, and NF-κB signaling in macrophages and VSMCs [43] (Table 1).

**Figure 1:**
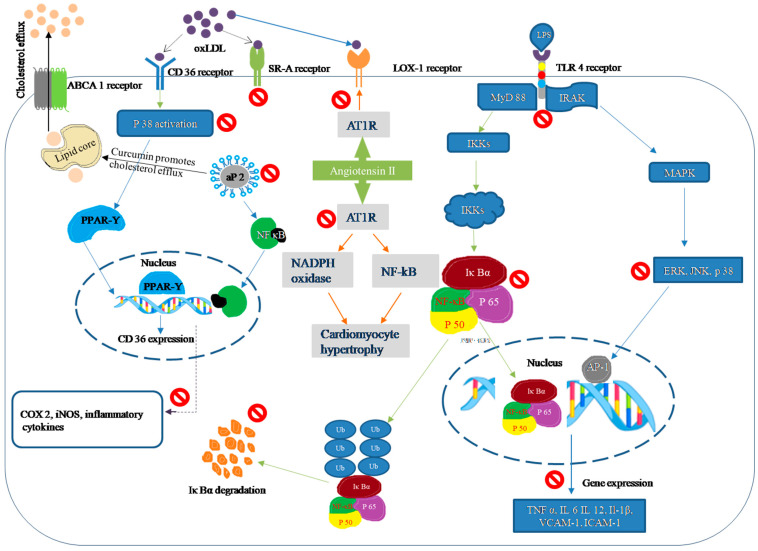
Pharmacological effects and mechanism of action of curcumin in atherosclerosis.

TLR4, an important signaling receptor, plays an important role in the pathogenesis of plaque formation and the development of atherosclerosis [73]. Furthermore, TLR4 activates a variety of signal transduction molecules as well as transcription factors. An important response of TLR4 activation is NF-κB and MAPK activation, which triggers nuclear transduction that simultaneously propels the gene expression profile of an inflammatory reaction. The amplified expression profile increases ROS production and the expression of inflammatory molecules, which causes the initiation of atherogenesis, leading ultimately to the clinically critical destabilization of atherosclerotic plaques [16]. Reports on curcumin supplementation fostering negative regulation not only on towards the TLR receptor but also on nuclear transduction molecules and inflammatory cytokines (TNF-α, IL-1β, VCAM-1, ICAM 1, etc.) are presented [74] (Figure 1).

Curcumin has also been shown to inhibit ligand-induced and ligand-independent dimerization at the receptor level. LPS induces activation of both MyD88 and TRIF-dependent signaling via the TLR4 receptor. Upon curcumin supplementation, TLR4 homodimerization was blocked [46], providing a novel mechanism for its anti-inflammatory effects. In a similar fashion, curcumin inhibits the NOD-like receptor (NLR) family, the pyrin domain containing 3 (NLRP3) inflammasome via suppressing TLR4/MyD88/NF-κB, the phosphorylation level of IkB-α, and purinergic 2X7 receptor (P2X7R) pathways in phorbol 12-myristate 13-acetate (PMA)-induced macrophages [55]. NLRP3 inflammasome is composed of a multiprotein complex having caspase and caspase 1 protein complex for apoptosis [75]. On NLRP3 complex stimulation, caspase-1 is activated, which cleaves the pro-forms of interleukin (IL)-1β and IL-18 into their mature forms. Once in fully mature form, IL-1β (a primary pro-inflammatory cytokine) mediates the development of atherosclerosis. Curcumin also inhibits VSMC migration by negatively regulating NLRP3 expression via an NF-κB-mediated response and decreasing IL-1 concentration [55].

In VSMCs, curcumin supplementation markedly reduces inflammatory responses induced by LPS acting at TLR4. LPS induced stimulation of TRL4 increases the phosphorylation of IκBα, NF-κB (p65), and MAPKs [59]. Concurrently, this increases the inflammatory cytokine expression profile of TLR4, MCP-1, iNOS, TNF-α, and NO production. In addition, Meng et al. (2013) [59] established that curcumin supplementation inhibits TLR4 activation and ERK1/2 and p38 MAPK phosphorylation, thereby preventing NF-κB nuclear translocation that mediates ROS production. Thus, inhibition of the expression profile may reduce atherosclerotic plaque formation and reduce inflammatory cell infiltration into the plaques. More recently, Zhang et al. [62] showed that curcumin inhibits aldosterone-induced production of CRP in VSMCs by reducing ROS production via limiting aberrant activation of the ERK1/2 signal pathway.

LDL is another important pathological entity that contributes to the development of atherosclerotic lesions. ROS modifies LDL, thereby producing Ox-LDL. An increase in Ox-LDL concentration in plasma has long been recognized as a key factor in atherosclerosis. Ox-LDL, rather than binding to LDL receptor, binds to scavenger receptors (SRs). The major SR is CD36 that recognizes ox-LDL [76]. After binding to CD36 on cell membrane, ox-LDL can also trigger CD36 expression via PPAR-γ pathway [77]. Specifically, PPAR-γ, once activated, dimerizes with the retinoid X receptor (RXR) and triggers PPAR-response element (PPRE)-containing genes, which ultimately increases CD36 expression, resulting in increased ox-LDL influx [78].

Cholesterol accumulation in macrophages results in foam cell formation and fatty streak development via upregulating the expression/activity of several receptors, such as SR-AI/II, SRBI, CD36, and LOX-1. In contrast, various efflux transporters play an active role via ATP-binding cassette (ABC) transporters ABCA1, ABCG1, and SR-BI to facilitate reverse cholesterol transport from macrophages [79]. Fatty acid-binding protein (FABP)-4 or adipocyte protein 2 (aP2) coordinates cholesterol trafficking (efflux) but is also known to activate an inflammatory response. Lack of aP2 protein complex changes the cholesterol composition in macrophages, which concurrently amplifies CD36 expression and enhances oxLDL influx [80]. This cascade creates a disease state, whereby macrophages induce the release of IL-1β, TNFα, ROS, and matrix metalloproteases coupled with the development of inflammation, cell migration, and plaque formation (Figure 1). Hence, genetic or pharmacological inhibition of aP2 and CD36 expression might offer potential remedies to atherosclerosis.

Several further lines of experimental evidence highlight the potent anti-atherogenic effects of curcumin (documented in Table 1). For example, Zhou et al. (2014) [36] demonstrated that curcumin treatment reduces the expression profile of oxLDL-induced thrombospondins-4 (THBS-4). THBS-4 was reported to influence important cellular responses such as cell migration, proliferation, and adhesion, leading to atherogenesis progression [81]. Curcumin further inhibits p38 MAPK activation and reduces PPAR-γ and CD36 expression in oxLDL-treated macrophages, leading to decreased foam cell formation [77]. In human umbilical vein endothelial cells (HUVECs), curcumin inhibits ROS production, NF-κB-dependent LOX-1 expression, and VCAM-1 and ICAM-1 expression. In addition, curcumin promotes NO production to confer vasodilatory effects [6,7]. Recent studies also suggest that curcumin could reduce oxidative stress, ER stress, and inflammatory response induced by acrolein (a toxin from tobacco smoke) and cytomegalovirus (CMV) infection in human endothelial cells [29,66]. The anti-inflammatory effects of curcumin is exerted through inhibiting COX-2 expression and prostaglandin production via reducing the phosphorylation of PKC, p38 MAPK, and cAMP response element-binding protein as well as inhibiting the HMGB1-TLRS-NF-κB signaling pathway [29,66]. The broad anti-inflammatory effects of curcumin underlie its effects on improving flow-mediated dilation in human subjects [82].

## 3. Atheroprotective Effects of Curcumin In Vivo

Numerous lines of experimental evidence (Table 2) support the actions of curcumin in reducing the cardiovascular risk associated with atherosclerosis.

## 4. Clinical Studies of Curcumin

Few clinical trials involving double-blind placebo-controlled studies and randomized controlled trials have been undertaken. A 12-week randomized placebo-controlled trial of 118 participants showed that curcumin treatment reduced the risk of developing acute cardiovascular events in people with type 2 diabetes and dyslipidemia [95]. Another randomized controlled research with 87 patients found that taking 1 g of curcumin for eight weeks lowered TC, TG, and HDL-c levels following nonalcoholic fatty liver infections [96]. On the other hand, curcumin lowered LDL-c and Apo B and increased Apo A1 and HDL-c levels in healthy people, indicating anti-atherosclerosis efficacy [97]. In coronary bypass graft, curcumin (4 g/day) reduced acute myocardial infarction and significantly decreased malondialdehyde levels [98]. Further, in patients with chronic obstructive pulmonary disease, curcumin (Theracurmin^®^ 90 mg/day for 24 weeks) reduced the level of the α1-antitrypsin–low-density lipoprotein (AT-LDL) complex, which promotes arteriosclerosis [99]. In another randomized trial, curcumin usage at 80 mg per day ameliorated dyslipidemia in patients with reduced serum TG, salivary amylase, and β-amyloid levels and increased plasma nitric oxide level after four weeks of study [100]. Likewise, in a double-blind placebo-controlled study, curcumin (200 mg) supplementation improved endothelial function measured by flow-mediated dilation (FMD), thus decreasing the risk of cardiovascular diseases [101]. In another pilot study, curcumin (500 mg/day for 12 weeks) de-stiffened arteries in young, obese men with aortic stiffness [102]. Studies with curcumin have potential limitations due to factors such as limited sample sizes; therefore, large-scale clinical trials are required to characterize the actual potential and identify the direct molecular targets of curcumin in treating atherosclerosis.

## 5. Conclusions and Perspectives

Substantial experimental evidence suggests that curcumin prevents endothelial dysfunction, smooth muscle cell proliferation and migration, and foam cell formation and modulates macrophage polarization. Curcumin also counteracts inflammatory response, supporting its potential application in atherosclerosis treatment. The anti-atherosclerotic properties of curcumin occur through suppressing inflammatory response by skewing macrophage polarization from M1 to M2 or by inducing M2 polarization through regulating TLR4/MAPK/NF-κB pathways in macrophages and secretion of interleukins (IL-4 and/or IL-13). Similarly, curcumin concurrently regulates the expression and activity of the lipid transporter expression (CD36, CD38, ABCA1, aP2, etc.) responsible for cholesterol uptake and efflux, thus maintaining cell homeostasis. In addition, curcumin lowers the circulating level of ox-LDL and blocks oxLDL elicited pro-atherogenic events by decreasing the expression of MCP-1 and THBS-4 via the p38 MAPK and NF-κB pathways [52]. Likewise, curcumin suppresses TLR4 expression and macrophage infiltration in aortic tissues and protects against atherosclerotic plaque formation [16]. A recent study has suggested that curcumin blocks LPA-induced MCP-1 expression via TGFBR1/ROCK signaling pathway [103].Additional studies are required to improve or add meaningful insights into our understanding of the mechanism(s) of action of curcumin against atherosclerosis, especially in the clinical setting. In addition, the development of novel drug delivery systems, such as the creation of curcumin nanomicelles [104,105], is critical for improving the oral bioavailability of curcumin which may contribute to its clinical efficacy [106].

## Figures and Tables

**Table 1:** In vitro evidence supporting the therapeutic potential of curcumin against atherosclerosis.

Experimental Model	Concentration Used	Outcomes and Possible Mechanisms of Action	References
U937 monocytes	0.01–1 µM	− Inhibit lipid peroxidation and inflammatory cytokine production under high glucose stimulated conditions	[44]
HMEC-1 cells	0.1–10 μM	− Reduce cell migration and viability and repress MMP-2, MMP-9, and VEGF expression − Upregulate miR-126 expression and inhibit PI3K/AKT and JAK2/STAT5 signal transduction	[42]
ANA-1 mouse macrophage cell line	5–25 μM	− Decrease THBS-4 expression as induced by oxLDL	[36]
RAW 264.7 macrophages		− Inhibit foam cell formation and CD36 expression level via blocking p38 MAPK phosphorylation	[34]
H9c2 rat cardiac myoblasts	5–40 μM	− Activate p38-MAPK and JNK signaling pathways − Promote apoptosis by chromatin condensation	[36]
Human monocytic THP-1 cells	7.5–30 μM	− Inhibit M1 macrophage polarization and cytokine production (IL-6, IL-12B, and TNF-α) and decrease TLR-4 expression − Inhibit ERK, JNK, p38, and NF-kB phosphorylation, exerting anti-inflammatory and anti-atherosclerotic activity	[43]
Human monocytic THP-1 cells	5–20 μM	− Reduce the influx of oxLDL in THP-1 cells − Suppress CD36 and aP2 expression	[45]
RAW264.7 macrophage	6.25 and 12.5 μM	− Increase cholesterol efflux via Apo-A1 and HDL in macrophages − Reduce oxLDL-induced cytokine production as well as M1 macrophage apoptosis − Upregulate CD36 and ABCA1 expression in M1 macrophages	[37]
Ba/F3 cells	10–20 μM	− Inhibit TLR4 dimerization at the receptor level − Inhibit the activation of MyD88 and TRIF-dependent pathways, thereby blocking NF-κB and IRF3 signaling	[46]
RAW264.7 macrophage	6.25–25 μM	− Inhibit the expression of M1 macrophage markers (i.e., iNOS, IL-1b, IL-6, and MCP-1) and upregulate IKBα expression	[47]
RAW264.7 macrophage	6.25–50 μM	− Upregulate the expression of M2 markers such as MMR, Arg-1, and PPAR-, as well as macrophage M2 polarization via IL-4 and/or IL-13 secretion.	[40]
RAW264.7 macrophage	6.25, and 25 nM	− Repress titanium (Ti) particle-induced inflammation via modulating macrophage M1 to M2 polarization	[34]
RAW264.7 macrophage	8–128 μM	− Inhibit lipid accumulation and the production of MCP-1, TNF-α, and IL-6	[48]
Mouse peritoneal macrophages	10–50 μM	− Reduce TLR4 expression and inhibit NF-κB activation	[16]
Human monocytic THP-1 cells	20–40 μM	− Inhibit HIF-1α-induced apoptosis and inflammation of macrophages via ERK signaling pathway	[49]
Bovine aortic endothelial cells (BAECs)	5–15 μM	− Inhibit the expression of ET-1mRNA in BAECs, which may influence the formation of atherosclerotic plaques	[50]
RAW264.7 macrophage	0.1–30 μM	− Repress IL-1β, IL-6, and TNF-α production	[51]
Human monocytic THP-1 cells	0–50 μM	− Attenuate MMP-9 and EMMPRIN expression via downregulation of NF-κB and p38 MAPK signaling	[52]
Human monocytic THP-1 cells	0 to 100 μM	− Inhibit MMP-9 and EMMPRIN expression via inhibiting AMPK and PKC pathway	[53]
Human monocytic THP-1 cells	10−20 μM	− Inhibit the PKC-δ/NADPH oxidase/ROS signaling and suppress matrix invasion	[54]
Human monocytic THP-1 cells	0–50 μM	− Suppress TLR4/MyD88/NF-κB and P2X7R signaling and inhibit inflammasome activation	[55]
THP1-derived macrophage foam cells	0–80 μM	− Promote cholesterol efflux via increased ABCA1 expression via AMPK-SIRT1-LXRa signaling pathway	[38]
Human monocytic THP-1 cells	5.0 µg/mL	− Increase macrophage apoptosis, thus indicating a novel son o-dynamic therapy for atherosclerosis	[56]
VSMCs	5–30 μM	− Suppress oxLDL induced MCP-1 expression via p38 MAPK and NF-κB signaling	[57]
H9c2 embryonic rat heart derived cells	5–15 μM	− Enhance DOX-induced cells apoptosis via Bcl-2 repression and increasing expression of caspase-8 and -9	[58]
VSMCs	5–30 μM	− Decrease the expression/level of MCP-1, TNF-α, NO, and ROS production − Suppress TLR4 activation and inhibit ERK1/2 and p38 MAPK phosphorylation	[59]
RAW264.7 macrophage	0–40 μM	− Inhibit MCP-1 production via the JNK and NK-κB signaling − Enhance cholesterol efflux via activating the LXR-α, ABCA1 and SR-BI pathway	[60]
3T3-L1 fibroblast cells	0–30 μM	− Inhibit MAPK phosphorylation by using Wnt/β-catenin signaling, which leads to 3T3-L1 cell differentiation into adipocytes	[61]
VSMCs	1.25–5 μM	− Inhibit CRP protein production by modulating ROS-ERK1/2 signaling	[62]
Endothelial cells	10^−5^ M	− Inhibit CD40 expression and inflammatory activity via miR-590-3p-dependent pathway	[63]
Cultured porcine coronary artery rings	5 μM	− Block superoxide anion production mediated by eNOS downregulation and reverse endothelial dysfunction	[64]
HUVEC cells	1, 10,100 μM	− Reduce E- and P-selectins expression and monocytes adhesion induced by PM10 (3 μg/cm^2^) and TiO2-NPs (10 μg/cm^2^) − Attenuate oxidative stress activation induced by PM10 particles and TiO2-NPs in endothelial cells	[65]
HUVEC cells	25 μM	− Inhibit COX-2 expression and prostaglandin production − Inhibit phosphorylation of PKC, p38 MAPK, and cAMP response triggering COX-2 expression	[66]
HUVEC cells	1–25 μM	− Suppress the expression profile of ROS species, LOX-1 receptor, and adhesion molecules (VCAM-1 and ICAM-1) − Inhibit IκBα degradation and NFκB nuclear translocation	[67]
HUVEC cells	2.5–100 μM	− Decrease TLR2 and TLR4 mediated inflammatory response − Inhibit adhesion molecules expression that reconcile monocyte adhesion and endothelial migration	[68]
HUVEC cells	3–30 μM	− Inhibit NF-κB activation via TNF-α − Suppress intracellular ROS production, monocyte adhesion, and JNK, p38, and STAT-3 phosphorylation − Attenuate expression profile of ICAM-1, MCP-1, and IL -8 at both mRNA and protein levels	[69]
VSMCs	20–40 μM	− Diminish phosphorylation of p-RhoA/p-MEK1/2 and NF-κB signaling	[70]
VSMCs	-	− Activate miR-22/SP1 signaling pathway and prevent proliferation and migration of VSMCs	[71]
VSMCs	12.5–50 μM	− Inhibit cholesterol accumulation via activating caveolin-1 expression that in turn negatively regulates SREBP-1 and prevents nuclear translocation	[72]
HUVEC cells	0.5–2 μM	− Inhibit HCMV replication and proliferation − Reduce intracellular ROS production and diminish inflammatory cytokine production − Downregulate HMGB1-TLR-NF-κB signaling	[29]
VSMCs	10–20 μM	− Reduce NO production by inhibiting IL-6 and TNF-expression − Upregulate PPAR-γ activity and attenuate VSMC proliferation	[34]
VSMCs	20 μM	− Inhibit cell migration by negatively regulating NLRP3 expression via NF-κB -mediated response and reduce IL-1β concentration	[26]

HMEC-1, human micro-vascular endothelial; PARP, poly(ADP-ribose) polymerase;MMR, macrophage mannose receptor; Arg-1, arginase-1; HIF-1α, hypoxia- inducible factor 1α; TGF-β, transforming growth factor beta; AMPK, AMP-activated protein kinase; PKC, protein kinase C; DOX, doxorubicin; ET-1, endothelin-1; PAR-γ, proliferator-activated receptor **γ**; LXR-α, liver X receptor α; SR-BI, scavenger receptor class B type I; JAKs, Janus activated kinases; iNOS, inducible nitric oxide synthase; MyD88, myeloid differentiation factor 88; P2X7R, purinergic 2X7 receptor; PKC, protein kinase C; AD, aldosterone, CRP, C-reactive protein; HUVEC, human umbilical vein endothelial cells; LOX-1, lectin-like oxidized LDL receptor-1; TEM, trans-endothelial migration; HMGB1, high mobility group box-1; MEK 1/2, mitogen-activated protein kinase kinase 1/2; JNK-c, Jun N-terminal Kinase.

**Table 2:** In Vivoevaluation of the pharmacological properties of curcumin against atherosclerosis.

In Vivo Experimental Model	Curcumin Concentration	Outcomes and Possible Mechanisms of Action	References
ApoE^−^/^−^ mice	0.1% *w*/*w*	− Downregulate TLR-4 expression − Reduce the expression of IL-1β, TNF-α, VCAM-1, and ICAM-1 and the activity of NF-κB − Inhibit macrophage infiltration, resulting in reduced atherosclerotic plaques and lesions development	[16]
Male New-Zealand rabbits	1.66 mg/kg body weight	− Reduce LDL propensity to lipid peroxidation − Decrease TC, TG, and phospholipids level in rabbits	[10]
New Zealand white male rabbits	10 mg/kg/week	− Reduce serum levels of TC, TG, and LDL-c − Decrease atherosclerotic lesions in the aortic arch	[35]
Ldlr^−^/^−^ mice	500–1500 mg/kg	− Reduce oxLDL uptake in HP-1 cells − Reduce the formation of fatty streaks and inhibit the expression of inflammatory cytokines, aP2, and CD36 − Repress the progression of steatohepatosis	[45]
Male Wistar rats	100 mg/(kg/d) curcumin	− Inhibit the expression profile of MMP-9, CD40L, TNF-α, and CRP, thereby improving the permeability of coronary artery	[83]
ApoE^−^/^−^ mice	200 mg/kg/d	− Modulate T helper cell (Th2) and regulatory T cells (Tregs) to recover the formed atherosclerotic lesions and plaque	[84]
Male Rabbits	0.2%	− Reduce the expression of CRP, ICAM1, VCAM1, and PCSK9 gene expression	[85]
ApoE/LDLR—doubleknockout mice	0.3 mg/perday	− Reduce TC and TG levels in blood − Reduce atherosclerotic lesion area and size	[20]
Male C57BL/6J (B6) mice	0.09 mg	− Prevent liver fat accumulation and development of atherosclerotic lesions − Improve hyperlipidemia state	[86]
ApoE^−^/^−^ mice	0.2%	− Reduce leukocyte adhesion and trans endothelial migration	[87]
LDLR^−^/^−^ mice	100 mg/kg	− Improve intestinal function against glucose intolerance − Reduce aortic lesion area	[88]
Sprague-Dawley rats	100 mg/kg body weight	− Inhibit the production of IL-6, TNF-α, IL-8, MCP-1, glucose, and glycosylated hemoglobin (HbA_1_)	[44]
Sprague-Dawley rats	0.2–5.0 mg/kg	− Inhibit the production of TNF-α, IL-1β,and MCP-1	[89]
Zebrafish	10% wt/wt	− Inhibit hyper cholesterolemic state and improve antioxidant activity	[90]
ApoE^−^/^−^ mice	15–25mg/kg/d	− Reduce LDL-c, TC, and TG − Decrease atherosclerotic plaque formation in the aorta and reduce lipid deposition in the liver and inflammatory damage in the heart, lung, and kidney	[29]
ApoE^−^/^−^ mice	10 mg/kg	− Reduce the formation of microvessel plaques, inhibit MMP-2 and -9 activity and regulate LDL-c metabolism	[59]
LDLR^−^/^−^ mice	0.02%*w*/*w*	− Decrease TC, TG, LDL-C, and Apo-B levels − Increase plasma HDL-c and liver Apo A-I expression − Inhibit HMG-CoA reductase, ACAT1, and ACAT2 expression	[91]
ApoE^−^/^−^ mice	40, 60, and 80 mg/kg/d curcumin	− Reduce lipocalin-2 (LCN2) biomarkers of atherosclerosis, present an anti-hyperlipidemic effect, and inhibit the inflammatory response	[92]
Male ICR mice	1–2mmol/kg/day	− Ameliorate dyslipidemia and hyperglycemia, reduce oxidative stress, and enhance antioxidant activity	[93]
ApoE^−^/^−^ mice	0.1% *w*/*w*	− Reduce TC accumulation in the aortas − Lower LDL-c level and decrease intestinal cholesterol absorption	[94]

VCAM-1, vascular cell adhesion molecule; ICAM-1, intracellular adhesion molecule; MMP, matrix metalloproteinase; Apo A-I, apolipoprotein A-I; Apo B, apolipoprotein B; HMG-CoA, 3-hydroxy-3-methyl-glutaryl-co-enzyme A reductase; ACAT, acyl-CoA/cholesterol acetyl transferases; TC, total cholesterol; TG, triglyceride; LDL-C, low-density lipoprotein cholesterol; HDL-C, high density lipoprotein cholesterol; CRP—creactive protein; MCP 1, monocyte chemoattractant protein 1.
